# Retraction Note: Dual versus mono antiplatelet therapy for acute non- cardio embolic ischemic stroke or transient ischemic attack, an efficacy and safety analysis - updated meta-analysis

**DOI:** 10.1186/s12883-020-02025-3

**Published:** 2020-12-17

**Authors:** Christessa Emille Que Albay, Frederick Gavril D. Leyson, Federick C. Cheng

**Affiliations:** Cardinal Santos Medical Center, 10 Wilson St. Greenhills West, 1502 San Juan City, NCR Philippines

**Retraction Note: BMC Neurol 20, 224 (2020)**

**https://doi.org/10.1186/s12883-020-01808-y**

The Editor has retracted this article [1]. This was an updated meta-analysis that included two additional studies; however, one of the studies included in the meta-analysis (Johnson et al. 2016, [14] in the article) should not have been included because it reports data comparing Ticagrelor with Aspirin (i.e. mono versus mono anti-platelet therapy) rather than data comparing Ticagrelor plus Aspirin with Aspirin alone (i.e. dual versus mono anti-platelet therapy). As this study has a large sample size its inclusion will have undermined the results and conclusions presented. Christessa Emille Que. Albay and Federick C. Cheng agree with this retraction. Frederick Gavril D. Leyson has not responded to correspondence from the Editor about this retraction.
